# Short-chain fatty acids act as metabolic and epigenetic regulators in CAR T cell therapy

**DOI:** 10.1016/j.omton.2026.201322

**Published:** 2026-08-12

**Authors:** Ming-Yang Ma, Chen-Xin Zhou, Xin-Yu Shi, Jian-Min Zhu, Cai-Wen Duan

**Affiliations:** 1 Pediatric Translational Medicine Institute, Shanghai Children’s Medical Center, Shanghai Jiao Tong University School of Medicine, Shanghai 200127, China

**Keywords:** CAR T, short-chain fatty acids, butyrate, pentanoate, tumor microenvironment, epigenetic reprogramming, mitochondrial metabolism, HDAC inhibition, *ex vivo* conditioning, solid tumors

## Abstract

Chimeric antigen receptor (CAR) T cell therapy has transformed the treatment of hematologic malignancies, but its efficacy in solid tumors remains constrained by poor infiltration, metabolic stress, and limited persistence. Short-chain fatty acids (SCFAs), particularly butyrate and pentanoate, offer a way to influence CAR T metabolism and chromatin state during manufacturing. Butyrate combines class I histone deacetylase inhibition with acetyl-CoA metabolism and AMP-activated protein kinase (AMPK)-associated restraint of mTORC1, whereas pentanoate can reinforce effector programs through mTOR signaling and a distinct TCA-ATP-citrate lyase carbon-routing pathway. Direct CAR T studies and clinical associations now support the biological relevance of both metabolites, although their effects depend on dose, exposure schedule, cell composition, and experimental context. Building on their complementary actions, we propose sequential butyrate-pentanoate conditioning, with early butyrate exposure used to support oxidative and progenitor-associated features and later pentanoate exposure used to reinforce effector function. This review develops the mechanistic basis for that strategy, defines the experiments needed to distinguish cooperation from antagonism, and considers its manufacturing and translational implications.

## Graphical abstract

**Figure:**
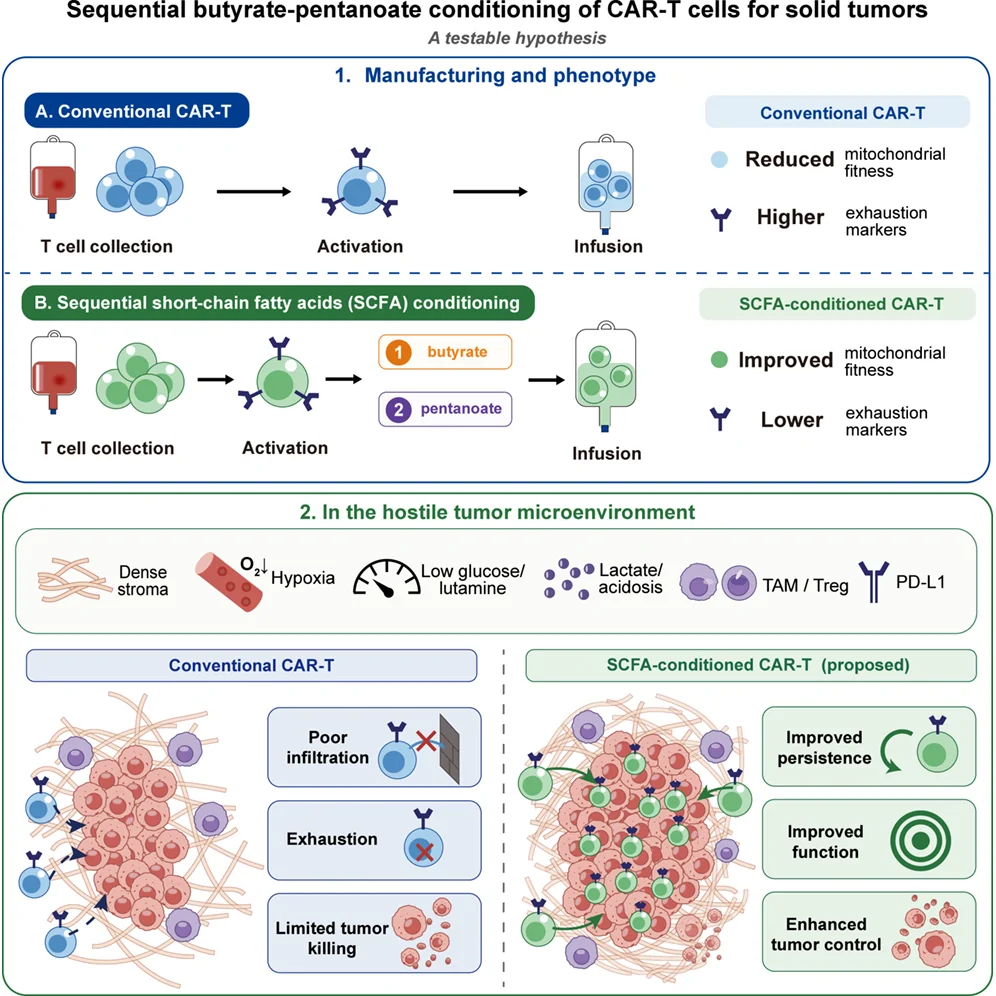

## Teaser

Ma and colleagues review how short-chain fatty acids shape T cell and CAR T metabolic and epigenetic states. They distinguish direct CAR T evidence from broader T cell findings and propose a testable sequential butyrate-pentanoate conditioning framework, outlining the comparisons and manufacturing controls needed to evaluate its potential in solid tumors.

## Introduction

Cancer represents one of the foremost causes of morbidity and mortality worldwide. According to GLOBOCAN 2022, approximately 20 million new cancer cases and 9.7 million cancer-related deaths were recorded globally, with projections indicating a 77% increase in incidence and a roughly 90% rise in mortality by 2050.^1^ This escalating burden underscores the urgent need for therapeutic strategies capable of achieving durable, broadly applicable antitumor efficacy.

**Figure 1:**
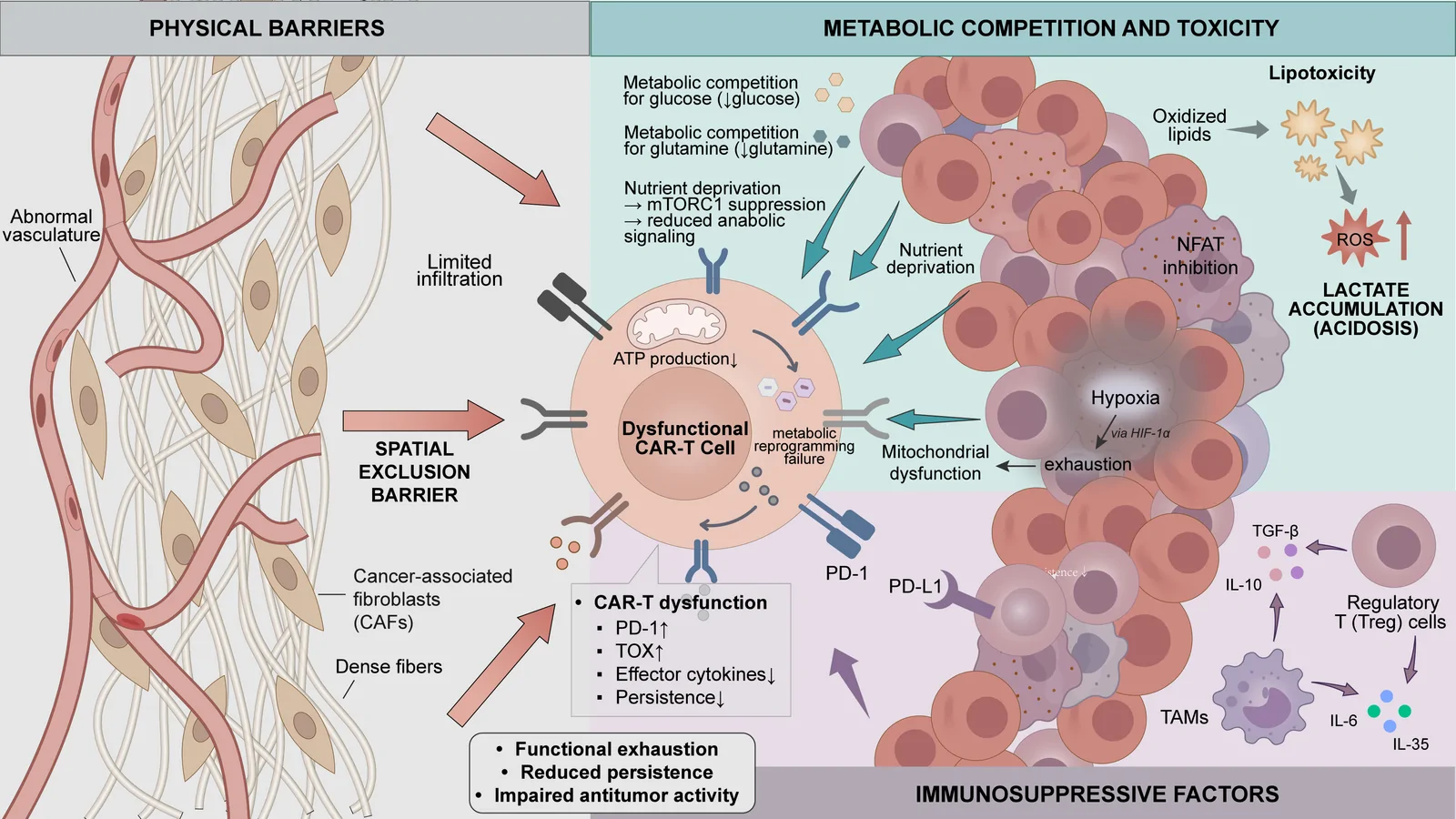
Major barriers within the tumor microenvironment limiting CAR T cell efficacy The diagram categorizes TME obstacles into three interrelated classes: (A) Physical barriers: abnormal vasculature, dense stromal fibers, and cancer-associated fibroblasts impose a spatial exclusion barrier that restricts CAR T cell trafficking and infiltration. (B) Metabolic competition and toxicity: tumor cell-driven depletion of glucose and glutamine suppresses mTORC1 and reduces anabolic signaling, while lactate accumulation, acidosis, hypoxia, and oxidized lipid-driven ROS cooperatively impair mitochondrial function. (C) Immunosuppressive networks: inhibitory cytokines, PD-1/PD-L1 checkpoint engagement, and recruitment of tumor-associated macrophages (TAMs) and regulatory T cells drive dysfunction programs, reduced effector cytokine secretion, and impaired persistence.

Among the emerging modalities of cancer immunotherapy, chimeric antigen receptor (CAR) T cell therapy has established a transformative clinical precedent in hematologic malignancies. The US Food and Drug Administration (FDA) has to date approved seven CAR T cell products for distinct hematologic indications,^2^ with pivotal trials demonstrating high complete remission rates in relapsed or refractory B cell malignancies and multiple myeloma.^3^^,^^4^^,^^5^^,^^6^^,^^7^ Despite this success, the extension of CAR T therapy to solid tumors has yielded consistently limited clinical outcomes.^8^ The mechanistic basis of this disparity lies in the multi-layered immunosuppressive architecture of the solid tumor microenvironment (TME), which imposes compounding barriers to CAR T cell infiltration, metabolic fitness, and functional persistence. These barriers can be broadly organized into three interrelated categories (Figure 1). First, physical barriers arising from dense stromal architecture and dysfunctional tumor vasculature restrict the spatial access of adoptively transferred T cells to tumor parenchyma.^9^^,^^10^^,^^11^ Second, metabolic competition and toxicity within the TME create a profoundly hostile nutritional landscape: highly glycolytic tumor cells deplete extracellular glucose and glutamine,^12^^,^^13^ depriving infiltrating CAR T cells of substrates essential for anabolic and bioenergetic programs. Concomitant accumulation of oxidized lipid species promotes dysfunctional T cell differentiation,^14^^,^^15^ while hypoxia, lactate accumulation, and acidosis cooperatively impair mitochondrial integrity and accelerate exhaustion.^16^ Third, immunosuppressive networks—comprising inhibitory cytokines, immunosuppressive myeloid populations including myeloid-derived suppressor cells (MDSCs) and tumor-associated macrophages (TAMs), and regulatory T cell-enriched niches—exert potent extrinsic constraint on CAR T activation and effector cytokine secretion.^17^^,^^18^^,^^19^^,^^20^^,^^21^ Collectively, these pressures converge to erode the metabolic and transcriptional programs required for sustained antitumor activity in solid tumor settings.

Host-microbiome interactions shape systemic immune programming and metabolic homeostasis. Among microbial metabolites, short-chain fatty acids (SCFAs), principally acetate, propionate, butyrate, and pentanoate, act as extracellular signals and intracellular substrates that link dietary fiber fermentation to adaptive immunity.^22^^,^^23^^,^^24^^,^^25^ Butyrate is one of the best-characterized SCFAs in T cells. It inhibits class I histone deacetylases, increases intracellular acetyl-CoA, and engages G protein-coupled receptors.^24^^,^^25^ Pentanoate has emerged as a distinct candidate. Direct CAR T studies show that it can alter engineered T cell metabolism and epigenetic state, while recent work links these effects to tricarboxylic acid-ATP-citrate lyase (TCA-ACLY) carbon routing.^26^^,^^27^ These findings extend the field beyond conventional T cell models and support direct evaluation across CAR designs and manufacturing conditions.

The convergence of these observations raises a compelling translational hypothesis: that SCFA-mediated reprogramming of CAR T cell metabolism and chromatin state during *ex vivo* manufacturing may confer the metabolic resilience and epigenetic fitness required to overcome TME-imposed dysfunction in solid tumor settings. The present narrative review synthesizes current evidence delineating how SCFAs modulate CAR T cell oxidative metabolism, mitochondrial bioenergetics, and effector transcriptional programs, and evaluates the translational implications for next-generation CAR T therapeutic development with butyrate as the best-characterized prototype and pentanoate as an emerging complement.

## CAR T construction and current therapeutic strategies

**Figure 2:**
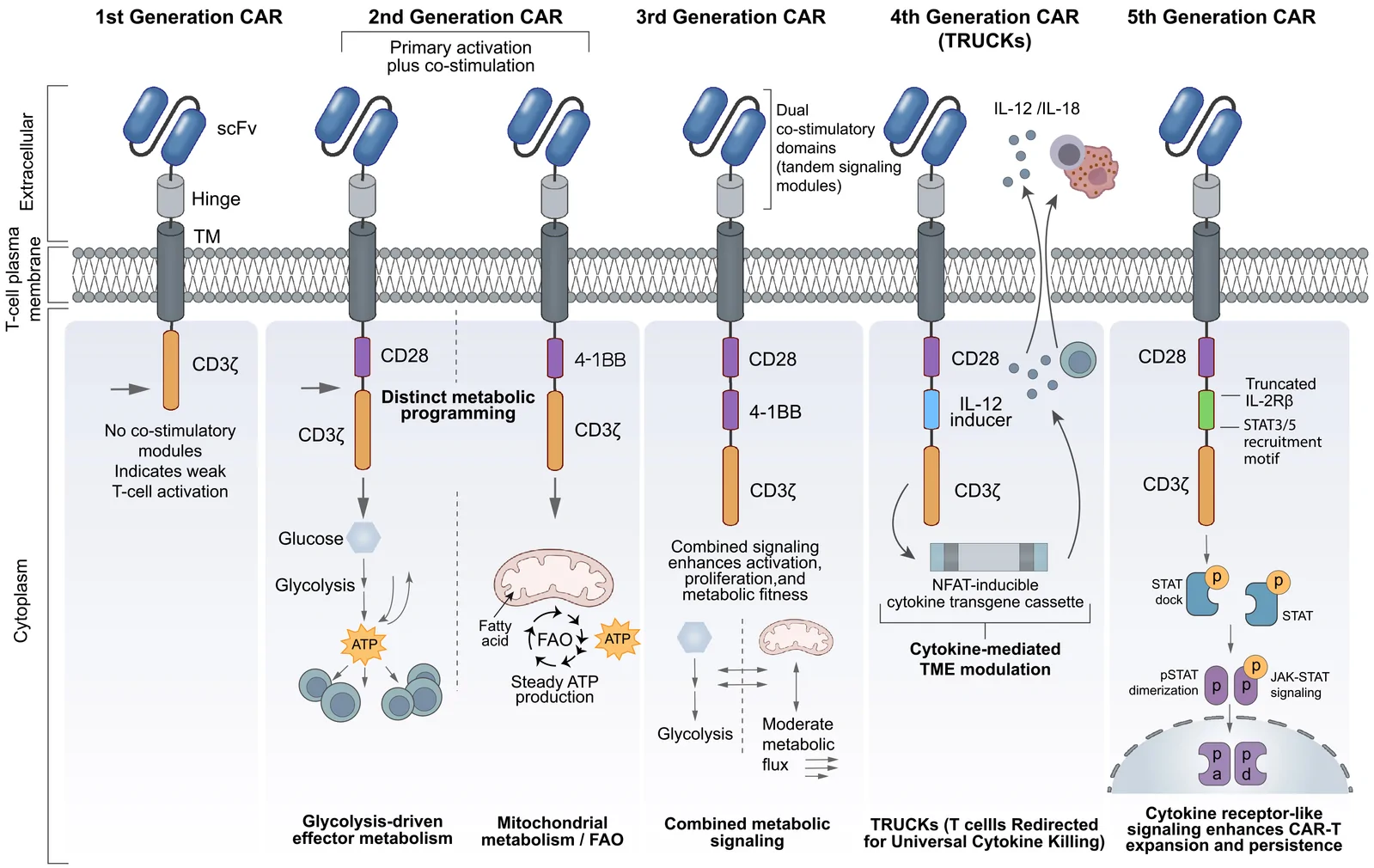
Development of CAR T cell generations and signaling architectures The schematic shows the progression from first-to-fifth-generation CAR constructs through the addition of costimulatory domains, inducible payloads, and cytokine-receptor motifs. Metabolic phenotype is determined mainly by the incorporated signaling modules rather than by generation number alone. CD28-containing CARs generally favor PI3K-AKT-mTOR signaling and glycolysis, whereas 4-1BB-containing CARs more often support mitochondrial biogenesis and oxidative phosphorylation. Fourth-generation TRUCKs release inducible immunomodulatory payloads, and fifth-generation CARs couple antigen recognition to cytokine-receptor signaling.

The functional architecture of a CAR determines the signaling inputs available to the engineered T cell and, consequently, its activation threshold, proliferative capacity, and metabolic phenotype. A canonical CAR molecule is organized into three modular components: an extracellular antigen-binding ectodomain, a transmembrane domain, and one or more intracellular signaling domains. The ectodomain typically consists of a single-chain variable fragment (scFv) fused to a structurally flexible hinge region, which positions the binding domain relative to the target antigen and influences both affinity and tonic signaling tone.^28^ Progressive refinements to this modular architecture have defined five generational classifications of CAR constructs, each introducing additional signaling capacity to address limitations identified in prior designs (Figure 2).^29^^,^^30^^,^^31^

First-generation CARs couple the antigen-recognizing scFv directly to the CD3 ζ intracellular signaling chain, providing TCR-proximal activation but lacking co-stimulatory input, which results in limited T cell proliferation, poor cytokine secretion, and inadequate persistence.^32^^,^^33^^,^^34^ Second-generation CARs addressed this deficiency by incorporating a single co-stimulatory domain, which is most commonly CD28 or 4-1BB (CD137), in tandem with CD3 ζ, substantially augmenting activation, clonal expansion, and *in vivo* durability.^35^^,^^36^^,^^37^ Third-generation constructs incorporate two co-stimulatory domains in series, with the intent of amplifying downstream signaling cascades, though the incremental benefit over second-generation designs remains context-dependent.^38^^,^^39^ Fourth-generation CARs, designated T cells redirected for universal cytokine killing (TRUCKs), are engineered with inducible transgenes encoding immunostimulatory cytokines or immune-modulatory payloads that are released upon CAR engagement^40^; these secreted factors act through autocrine and paracrine mechanisms to amplify cytotoxic programs and remodel the surrounding immune milieu.^41^^,^^42^^,^^43^ Fifth-generation CARs represent the current frontier of structural innovation, integrating truncated cytokine receptor signaling motifs that couple antigen recognition to Janus kinase/signal transducer and activator of transcription (JAK/STAT) pathway activation, enabling antigen-driven transcriptional reprogramming and enhanced autonomous proliferative signaling.^44^^,^^45^

A critical but underappreciated dimension of CAR structural design is its direct influence on T cell metabolic programming. Costimulatory domains are not metabolically neutral; rather, they bias the intracellular signaling environment in ways that durably orient CAR T cells toward distinct metabolic states.^46^^,^^47^^,^^48^^,^^49^^,^^50^ CD28-based CARs potently activate the PI3K-Akt-mTOR axis, driving upregulation of glucose transporters and committing cells to aerobic glycolysis as the dominant bioenergetic program.^51^ This glycolytic orientation confers a proliferative and cytokine-secretory advantage during early effector responses,^52^ but at the cost of reduced oxidative reserve and an accelerated trajectory toward terminal differentiation. Kawalekar et al. directly quantified this trade-off, demonstrating that CD28 ζ CAR T cells exhibit significantly higher glycolytic flux but substantially lower spare respiratory capacity (SRC) relative to their 4-1BB ζ counterparts.^53^ In contrast, 4-1BB co-stimulation preferentially engages NF-κB-associated signaling networks and promotes mitochondrial biogenesis, resulting in greater oxidative phosphorylation (OXPHOS) capacity, enhanced mitochondrial mass, and improved long-term persistence.^54^^,^^55^^,^^56^ Relative to CD28-based designs, 4-1BB CAR T cells exhibit reduced glycolytic dependence, broader metabolic flexibility, and a memory-biased differentiation trajectory that may confer a functional advantage under the glucose-limited conditions characteristic of solid tumors.^51^ Among other co-stimulatory domains, tumor necrosis factor receptor (TNFR) superfamily members including OX40 and CD27 have been reported to favor pro-survival signaling and oxidative metabolic programs, although systematic metabolic profiling of these domains remains limited.^57^ Inducible T cell costimulator (ICOS)-containing CARs engage PI3K through a signaling complex distinct from the CD28-p85 interaction and may generate a less glycolysis-skewed metabolic phenotype in defined T cell subsets, though the mechanistic basis and functional consequences of this distinction require further characterization.^58^

Taken together, the accumulated evidence establishes costimulatory domain selection as a primary determinant of intrinsic metabolic orientation in CAR T cells. This relationship carries particular clinical relevance in the solid tumor context, where chronic antigen exposure, glucose scarcity, and oxidative stress impose sustained pressure on infiltrating effector cells. Constructs that imprint glycolytic dominance may be disproportionately disadvantaged under these conditions, whereas those that support mitochondrial fitness and metabolic flexibility may better sustain effector function over time. This reasoning motivates a systematic examination of the specific metabolic constraints operating within the TME and the mechanisms by which they erode CAR T cell function. This is the focus of the following section.

## Major metabolic challenges in CAR T cell therapy

### Glucose metabolism: Glucose scarcity in the TME constrains CAR T effector functions

Glucose serves as both a central metabolic substrate and a regulatory signal during T cell activation. Upon antigen engagement, TCR and co-stimulatory cues rapidly activate the PI3K-Akt-mTORC1 axis, enhancing glucose uptake via GLUT1 (glucose transporter 1) and GLUT3 (glucose transporter 3) and driving aerobic glycolysis to sustain the anabolic demands of clonal expansion.^51^^,^^59^^,^^60^^,^^61^^,^^62^ mTORC1 further stabilizes HIF-1α, reinforcing glycolytic gene expression and supporting effector-lineage differentiation.^51^^,^^63^ Beyond its role as fuel, glucose metabolism generates critical metabolic signals: glyceraldehyde-3-phosphate dehydrogenase (GAPDH) represses IFN-γ mRNA translation when glycolytic flux is low,^52^ and phosphoenolpyruvate (PEP) sustains TCR signaling by enhancing Ca^2+^ flux and promoting nuclear factor of activated T cells (NFAT) nuclear translocation.^64^^,^^65^ Taken together, glucose availability is intricately coupled to cytokine production, cytotoxicity, and T cell fate decisions—positioning glucose not merely as a bioenergetic substrate but as a licensing signal for full effector programming.

In circulation, adequate glucose availability generally supports CAR T clonal expansion and effector function following infusion, underpinning the early antitumor responses documented in hematologic malignancies. However, this metabolic dependence becomes a liability upon tumor infiltration. Within the TME, glucose is severely depleted by the competing glycolytic activity of tumor cells and by nutrient consumption among stromal and myeloid populations.^12^^,^^16^ Lactate, a byproduct of tumor aerobic glycolysis, accumulates concomitantly, suppressing NFAT signaling and cytokine production through mechanisms independent of direct glucose limitation. Hypoxia further compounds this hostile landscape: while HIF-1α stabilization in tumor cells supports glycolytic adaptation, it paradoxically impairs mitochondrial function in infiltrating T cells and engages MYC-regulated dysfunction programs.^16^ Collectively, these pressures impair mTORC1 signaling, erode glycolytic capacity, and propel CAR T cells toward a metabolically exhausted phenotype. Emerging frameworks have reframed glucose, amino acids, and lipids as signals that license or suppress T cell immunity in concert with classical antigen and co-stimulatory inputs that establish metabolic flexibility, rather than glycolytic dominance, as a prerequisite for durable CAR T function in solid tumors.^66^^,^^67^

### Amino acid metabolism: Context-dependent benefits but poor durability under chronic TME restriction

While glucose fuels the energetic demands of T cell activation, amino acid metabolism provides the biosynthetic substrates and signaling cues that govern T cell fate and differentiation. Upon activation, T cells undergo marked upregulation of glutamineuptake mediated primarily by ASCT2 (alanine, serine, and cysteine transporter 2) and LAT1 (large neutral amino acid transporter 1),^68^^,^^69^ with additional contributions from γ-aminobutyric acid transporter (GAT) and betaine/GABA transporter (BGT) family transporters.^70^^,^^71^^,^^72^ Intracellularly, glutamine is converted by glutaminase to glutamate and subsequently to α-ketoglutarate (α-KG), fueling TCA anaplerosis and supporting OXPHOS while providing carbon skeletons for nucleotide, amino acid, and lipid biosynthesis during clonal expansion.^73^^,^^74^^,^^75^ mTORC1 serves as a central integrator of amino acid availability and anabolic growth programs^76^^,^^77^: lysosomal sensing of glutamine and leucine via the Ragulator-Rag GTPase complex activates mTORC1, which reinforces effector differentiation while suppressing memory-associated transcriptional programs.

Although transient amino acid restriction has been explored as a manufacturing strategy, short-term glutamine limitation during *ex vivo* expansion can attenuate mTORC1 activity and bias CAR T cells toward stemness-associated phenotypes.^78^ Such benefits are, however, difficult to sustain upon TME infiltration. Chronic glutamine depletion within the tumor stroma suppresses protein synthesis, reduces mTORC1 activity below the threshold required for effector maintenance, and engages stress-adaptive pathways.^79^^,^^80^ Competition for amino acids among tumor cells, cancer-associated fibroblasts, and myeloid populations imposes spatial and temporal heterogeneity in substrate availability,^81^^,^^82^ further eroding the reliability of amino acid pathways as a durable energetic or signaling resource. The central challenge, therefore, is to preserve CAR T biosynthetic capacity while circumventing the chronic amino acid-mTORC1-driven differentiation programs that accelerate exhaustion.^66^^,^^67^

### Lipid metabolism: Introducing SCFAs as promising substrates within the TME

Lipid metabolism governs CAR T persistence by shaping mitochondrial bioenergetics, SRC, and tolerance to oxidative stress. Resting and memory T cells rely predominantly on fatty acid oxidation (FAO) to maintain OXPHOS and mitochondrial mass, properties associated with longevity and recall responses.^83^ Under nutrient stress, infiltrating T cells can partially shift toward lipid catabolism as an adaptive response.^84^^,^^85^ Although oxidative lipid metabolism is beneficial in principle, the lipid landscape encountered within solid tumors is profoundly maladaptive. The TME is characterized by accumulation of oxidized low-density lipoproteins (oxLDLs), cholesterol-rich species, and polyunsaturated fatty acids susceptible to peroxidation.^12^^,^^86^ Uptake of oxidized lipids activates stress pathways, promotes lipid peroxidation, and suppresses cytokine secretion.^15^ Excess lipid accumulation also impairs mitochondrial β-oxidation and precipitates lipotoxicity, ultimately driving functional exhaustion.^14^

This creates a therapeutic paradox: CAR T cells require oxidative metabolism to persist in solid tumors, yet the endogenous lipid substrates abundant in the TME are broadly immunotoxic. SCFAs—principally acetate, propionate, butyrate, and pentanoate—represent a mechanistically distinct class of alternative substrates that may resolve this paradox: unlike long-chain fatty acids, SCFAs support mitochondrial OXPHOS while simultaneously engaging signaling and epigenetic programs that regulate T cell differentiation and persistence, properties whose mechanistic basis is examined in detail in the following section (Table 1).^22^^,^^26^^,^^27^

**Table 1:** Metabolic pathway characteristics and implications for CAR T cell therapy in the TME

Metabolic class	Description		Analysis	
	Key findings	Remarks	Potential strategies	Limitations and risks
Glucose metabolism	drives CAR T clonal expansion and effector cytokine production; GAPDH-mediated IFN-γ mRNA regulation and PEP-dependent NFAT nuclear translocation couple glycolytic flux directly to effector output	indispensable for early activation; glycolytic capacity licenses downstream cytokine secretion and effector programming	optimize *ex vivo* glucose availability to maximize effector fitness prior to infusion	depleted by competing tumor glycolysis and hypoxia in the TME; lactate accumulation suppresses NFAT signaling; impaired mTORC1 activity promotes exhaustion and reduces persistence
Amino acid metabolism	glutamine uptake via ASCT2 and LAT1 fuels TCA anaplerosis; Ragulator-Rag-dependent mTORC1 activation supports effector differentiation and biosynthetic programs	transient glutamine restriction during *ex vivo* expansion attenuates mTORC1 and can enrich stemness-associated phenotypes	short-term amino acid modulation during manufacturing to bias CAR T cells toward memory-like or progenitor states	chronic TME depletion suppresses protein synthesis and mTORC1 below effector maintenance threshold; substrate availability is spatially heterogeneous; long-term dependence is poorly durable
Long-chain fatty acids	FAO sustains mitochondrial OXPHOS and spare respiratory capacity; underpins memory T cell longevity and recall capacity	beneficial under controlled conditions; functional advantage contingent on lipid quality, which is broadly maladaptive in the TME	may support oxidative metabolism and long-term persistence under defined *ex vivo* conditions	TME accumulation of oxidized lipids (oxLDL, peroxidized PUFAs) drives lipotoxicity, ROS-mediated mitochondrial dysfunction, and impaired cytokine secretion; unregulated uptake accelerates exhaustion
Short-chain fatty acids (SCFAs)	provide alternative OXPHOS substrates; butyrate inhibits class I HDACs and expands the nuclear acetyl-CoA pool; pentanoate engages a TCA-ACLY-dependent nuclear carbon routing pathway not shared by C2-C4 SCFAs	couple metabolic substrate use with chromatin regulation; butyrate and pentanoate engage partly distinct pathways, providing a rationale for testing sequential exposure	*ex vivo* metabolic-epigenetic conditioning; sequential butyrate-pentanoate protocol proposed to prime oxidative fitness and subsequently arm effector programs prior to infusion	narrow therapeutic window; effects depend on dose, timing, and T cell subset; regulatory T cell induction is a concern in CD4+ cells; evidence remains limited across CAR designs and independent solid-tumor models

ACLY, ATP-citrate lyase; ASCT2, alanine-serine-cysteine transporter 2; FAO, fatty acid oxidation; HDAC, histone deacetylase; LAT1, large neutral amino acid transporter 1; NFAT, nuclear factor of activated T cells; OXPHOS, oxidative phosphorylation; oxLDL, oxidized low-density lipoprotein; PEP, phosphoenolpyruvate; PUFA, polyunsaturated fatty acid; ROS, reactive oxygen species; SCFA, short-chain fatty acid; TCA, tricarboxylic acid; TME, tumor microenvironment; Treg, regulatory T cell.

Taken together, the challenges associated with glucose, amino acid, and long-chain lipid metabolism delineate a shared set of metabolic constraints that limit CAR T function across solid tumor settings. SCFAs therefore emerge as a distinct metabolic class capable of simultaneously supporting oxidative metabolism and regulating signaling and epigenetic programs, making them especially attractive candidates for CAR T metabolic reprogramming.

## SCFAs act as both metabolic and epigenetic regulators

SCFAs influence immune cells through three principal routes: G protein-coupled receptor signaling,^87^^,^^88^^,^^89^ class I histone deacetylase inhibition,^25^^,^^26^^,^^90^ and the provision of acetyl-CoA or acyl-CoA intermediates for oxidative metabolism and histone modification.^91^^,^^92^^,^^93^ When these processes occur concurrently, increased acetyl-CoA can support histone acetyltransferase activity while histone deacetylase (HDAC) inhibition limits removal of the resulting marks. This coupling provides a plausible route from short-term metabolic exposure to more persistent changes in chromatin accessibility. It also distinguishes SCFAs from interventions that act primarily on either substrate metabolism or HDAC activity alone.

Although these metabolites engage overlapping pathways, individual SCFAs differ in their biological potency, metabolic utilization, and immunological consequences. Acetate has relatively weak HDAC-inhibitory activity but can supply acetyl-CoA through ACSS1 and ACSS2 during nutrient restriction.^91^^,^^92^ Propionate is a stronger HDAC inhibitor than acetate and, after conversion to propionyl-CoA, can activate the acetyltransferase p300.^90^^,^^94^ Butyrate combines class I HDAC inhibition with mitochondrial oxidation and has the broadest evidence base in T cells and CAR T models.^22^^,^^25^^,^^26^^,^^78^ Pentanoate can enter the TCA cycle through acetyl-CoA and succinyl-CoA and may supply nuclear acetyl-CoA through ACLY.^27^ These differences provide a rational basis for selecting or combining SCFAs according to the intended metabolic and epigenetic effect.

### Butyrate-mediated metabolic reprogramming

Butyrate enters T cells primarily via MCT1 (SLC16A1) and SMCT1 (SLC5A8) and is rapidly converted to acetyl-CoA, expanding the substrate pool for TCA cycle anaplerosis and histone acetyltransferase (HAT)-mediated histone acetylation.^95^ Extracellularly, butyrate engages GPR43/FFAR2 and GPR41/FFAR3, initiating downstream signaling that modulates metabolic programs independently of canonical antigen stimulation.^96^ In conventional CD8^+^ T cells, physiological butyrate concentrations drive a coordinated metabolic shift: enhanced mitochondrial biogenesis, elevated FAO, increased SRC, and reduced glycolytic reliance, collectively representing a metabolic phenotype associated with T cell memory and long-term persistence.^22^^,^^26^^,^^97^ These effects are mediated through transcriptional upregulation of PGC-1α, CPT1A, and mitochondrial transcription factor A (TFAM), which increase mitochondrial mass and oxidative capacity, and through butyrate-driven AMPK activation, which restrains mTORC1 to prevent terminal effector differentiation while preserving biosynthetic competence.^98^ Importantly, this metabolic shift is not solely a consequence of HDAC inhibition: the direct bioenergetic entry of butyrate-derived acetyl-CoA into the TCA cycle independently fuels OXPHOS, establishing butyrate as a bifunctional agent whose metabolic and epigenetic activities mechanistically reinforce each other.

Direct CAR T evidence supports biological activity of butyrate and pentanoate in several systems, while also showing that the response is context-dependent.^26^^,^^27^^,^^78^^,^^99^ Conversely, an intestinal adenocarcinoma-on-chip study found that butyrate and propionate reduced anti-ROR1 CAR T infiltration, cytotoxicity, and cytokine release while increasing regulatory and exhaustion-associated markers.^100^ Butyrate enhanced activation or antitumor activity in murine anti-ROR1, human CD19, and mesothelin (MSLN) CAR T models,^26^^,^^78^^,^^101^ and pentanoate improved function in several CAR T models.^27^ In an 84-patient non-Hodgkin lymphoma cohort, lower pre-infusion serum butyrate was associated with poorer progression-free survival, and matched experiments showed increased CD19 CAR T cytotoxicity after butyrate exposure.^101^ A separate multicenter study linked antibiotic-associated loss of microbial metabolic output to poorer CAR T outcomes.^102^ Together, these findings support SCFAs as biologically relevant modifiers of CAR T function, with metabolite, dose, exposure schedule, CAR design, and model context determining the direction and magnitude of the effect.

Optimizing these metabolic benefits requires operating within a narrow therapeutic window. First, dose and exposure duration are critical: low-to-moderate concentrations during manufacturing imprint mitochondrial fitness and reduce mTORC1-driven terminal differentiation without compromising viability, whereas high concentrations or prolonged exposure suppress mTORC1 excessively, impair proliferative capacity, and may engage apoptotic pathways.^26^^,^^99^^,^^103^ Second, route of administration is not interchangeable with *ex vivo* conditioning: systemic SCFA elevation affects a broad range of immune and non-immune cell populations, can enhance Treg accrual and dampen effector responses in some settings, and is substantially harder to calibrate than manufacturing-phase conditioning.^23^^,^^104^^,^^105^^,^^106^ These constraints position butyrate as a precision metabolic adjuvant for the *ex vivo* context, where dosing, timing, and cell population specificity can be rigorously controlled.^107^^,^^108^

### Butyrate-driven epigenetic remodeling

T cell exhaustion involves progressive chromatin remodeling, with reduced accessibility at loci associated with progenitor and effector functions and persistent accessibility at regulatory elements linked to inhibitory programs.^109^^,^^110^^,^^111^^,^^112^^,^^113^ Thymocyte selection-associated high mobility group box (TOX) and related signaling networks contribute to this reorganization,^113^^,^^114^^,^^115^ whereas TCF-1 supports progenitor-like states and recall capacity.^116^^,^^117^^,^^118^ As these configurations become increasingly resistant to reversal, the manufacturing phase provides a practical window in which to influence differentiation before chronic antigen exposure.^119^

Butyrate can influence this trajectory through class I HDAC inhibition and increased acetyl-CoA availability.^25^^,^^26^^,^^90^^,^^120^^,^^121^^,^^122^^,^^123^ In conventional T cells, SCFA exposure increases histone acetylation at effector-associated loci and alters cytokine responsiveness.^24^^,^^25^^,^^26^ Direct CAR T studies further show shifts toward memory-associated phenotypes and enhanced effector function after butyrate exposure.^26^^,^^78^^,^^101^ These findings support the use of butyrate during manufacturing to bias chromatin and differentiation before persistent exhaustion is established. The locus specificity and durability of this state are likely to depend on CAR design and culture conditions.

An emerging dimension of this epigenetic reprogramming involves the acetyl-CoA metabolism of the nucleus itself. ACSS2 and ACLY act as hubs that convert metabolic substrates into the acetyl units deposited on chromatin. Recent evidence suggests that manipulating nuclear acetyl-CoA metabolism can improve antitumor responses in dysfunctional CD8^+^ T cell states, pointing to SCFA-based conditioning and nuclear acetyl-CoA engineering as potentially synergistic strategies for maximizing epigenetic reprogramming gains.^124^

### Pentanoate: A distinct metabolic-epigenetic axis in CAR T engineering

Pentanoate occupies a distinct position among SCFAs because its C5 carbon skeleton can enter the TCA cycle through both acetyl-CoA and succinyl-CoA. In direct CAR T experiments, pentanoate-derived citrate was linked to nuclear acetyl-CoA production through ACLY, and ACLY inhibition reduced the associated cytotoxic benefit.^27^ This work connects mitochondrial carbon use with histone acetylation in engineered T cells rather than relying only on conventional T cell extrapolation. Independent replication will determine how broadly the pathway applies, but the available functional evidence already supports pentanoate as a CAR T engineering metabolite. If conserved across platforms, the TCA-ACLY route would complement HDAC inhibition and provide a mechanistic basis for staged use with butyrate.^124^^,^^125^^,^^126^

Functionally, pentanoate inhibits class I HDACs and enhances mTOR activity, augmenting effector molecule production including IFN-γ, TNF-α, and CD25.^26^^,^^127^ Critically, these effects have now been demonstrated in CAR T cell models directly: recent work has shown that pentanoate reprograms CAR T cell metabolism and epigenetic state, and enhances antitumor function in engineered T cell systems,^26^^,^^27^ moving the evidence base beyond extrapolation from conventional, non-engineered T cells. The extent to which the magnitude of pentanoate’s epigenetic and metabolic effects is modulated by CAR-mediated tonic signaling and by the specific costimulatory domain incorporated, which establishes different baseline mTOR tone in CD28 ζ versus 4-1BB ζ constructs, remains an open calibration question that direct comparative studies are needed to resolve. Similarly, the concentration range at which pentanoate supports progenitor-like T cell fates without engaging the anti-proliferative ceiling associated with high-dose class I HDAC inhibition requires empirical determination in each manufacturing context.^26^^,^^27^

Direct experiments show that pentanoate-conditioned CAR T cells can reduce exhaustion-associated features, increase progenitor and effector programs, and improve tumor control in selected hematologic and solid-tumor models.^27^ These data establish that pentanoate can act directly during CAR T engineering. Together with the distinct metabolic actions reported for butyrate, they provide a mechanistic basis for testing whether sequential exposure can combine early oxidative and progenitor-associated programming with later effector reinforcement.^26^^,^^27^^,^^127^

Despite this mechanistic progress, several limitations must be addressed before clinical translation. The therapeutic window for each SCFA should be empirically calibrated across different CAR constructs, T cell subset compositions, and manufacturing protocols; generalizing concentration optima from one system to another carries substantial risk of under- or over-dosing.^103^^,^^107^

SCFA-mediated HDAC inhibition in CD4^+^ T cells is a consequential manufacturing risk. Butyrate and propionate can promote FOXP3 expression and regulatory T cell differentiation at concentrations that also alter CD8^+^ cells.^23^^,^^25^^,^^127^^,^^128^^,^^129^ Because clinical products contain variable CD4:CD8 ratios, the magnitude of this effect is likely to differ across donors. CD8^+^ enrichment could reduce regulatory T cell induction but would also remove helper cells, while lower-dose butyrate may preserve part of the CD8^+^ benefit with less pressure on CD4^+^ cells.^26^^,^^99^ These trade-offs should be measured directly during process development. FOXP3^+^CD25^+^ frequencies should be monitored together with expansion yield, transduction efficiency, phenotype, and potency. Evidence now includes direct CAR T studies in hematologic and solid-tumor models,^26^^,^^27^^,^^78^^,^^101^ but independent validation across CAR designs and immunocompetent solid-tumor systems remains important.^8^^,^^31^

### Reconciling divergent mTOR modulation: A proposed sequential conditioning framework for butyrate-pentanoate co-programming

Butyrate and pentanoate have produced different mTOR-related effects across experimental systems.^26^^,^^127^^,^^130^^,^^131^^,^^132^^,^^133^ Butyrate-associated AMPK signaling has been linked to mTORC1 restraint, whereas pentanoate increased S6 phosphorylation and effector-molecule expression in CAR T models.^26^^,^^127^ Because these findings were obtained in different models, the outcome of simultaneous exposure could be additive, neutral, or antagonistic. This uncertainty does not weaken the rationale for combination; it argues for temporal separation and direct comparison.

We therefore propose a temporally staged conditioning model that follows the differentiation sequence of CAR T manufacturing. During early *ex vivo* expansion, butyrate-mediated mTORC1 restraint may limit premature effector differentiation, support mitochondrial biogenesis, and preserve TCF-1-associated progenitor features.^134^^,^^135^ Butyrate would serve as the metabolic and epigenetic primer in this phase. Later pentanoate exposure could reinforce mTOR-associated effector transcription after those features have been established.^26^^,^^136^ Its proposed TCA-ACLY carbon-routing pathway could add acetyl-CoA supply at accessible effector loci, providing a rationale for staged rather than simultaneous exposure.^124^^,^^137^

This sequential model is conceptually supported by the distinct mechanistic targets through which each SCFA is proposed to engage the mTORC1 axis. Butyrate-driven mTORC1 suppression appears to involve AMPK-associated metabolic sensing during oxidative utilization, together with HDAC-linked transcriptional programs that favor mitochondrial fitness and restrained effector differentiation.^138^^,^^139^^,^^140^ Pentanoate, by contrast, appears to support mTOR-associated effector output in parallel with enhanced citrate production and ACLY-dependent acetyl-CoA routing, suggesting that its dominant intracellular signal may reflect anabolic carbon sufficiency rather than energetic restraint.^125^^,^^126^ The mechanistic entry points of the two SCFAs are therefore potentially non-overlapping, but this characterization is itself inferential, derived from comparing independent experimental systems rather than from direct head-to-head measurement in the same cellular context. Whether the two effects are genuinely sequential modulators of distinct mTORC1-dependent transcriptional programs, or whether they converge on shared regulatory nodes in ways that would generate antagonistic rather than additive outcomes, cannot be determined without experiments that apply both agents in sequence within a single CAR T manufacturing system and directly measure chromatin-level and functional outputs at each stage.

**Figure 3:**
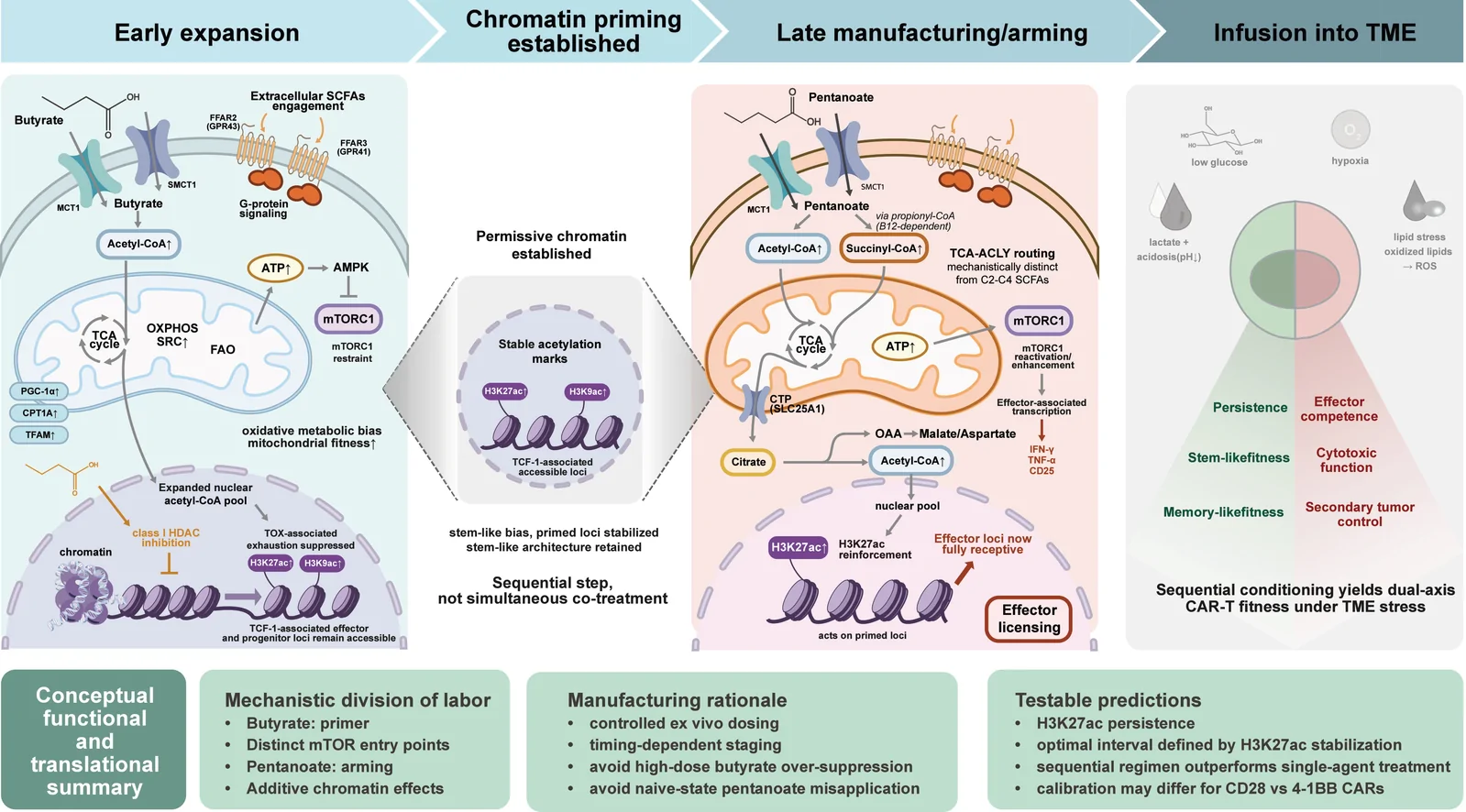
Sequential butyrate-pentanoate conditioning framework across CAR T manufacturing stages The schematic depicts early butyrate exposure linking class I HDAC inhibition and acetyl-CoA metabolism with oxidative and progenitor-associated programming, followed by later pentanoate exposure linking mTOR-associated signaling and TCA-ACLY carbon routing with effector function. The post-infusion image shows the proposed balance between persistence and cytotoxic activity under TME-imposed metabolic stress.

The sequential model yields three discriminating predictions. First, butyrate-associated chromatin and metabolic features should persist after pentanoate exposure rather than be erased by mTOR reactivation. Second, the interval between treatments should be defined from the kinetics of these features after butyrate withdrawal. Third, the sequence should outperform the better single agent in tumor-rechallenge assays. Vehicle, each single agent, simultaneous exposure, and both sequence orders should be compared under matched dose, duration, and washout conditions. Testing both CD28- and 4-1BB-based CARs will show whether baseline costimulatory signaling changes the response^53^^,^^54^^,^^55^^,^^56^^,^^141^^,^^142^ (Figure 3).

### Positioning SCFA conditioning among other CAR T engineering strategies

SCFA conditioning should be considered alongside other strategies used to preserve CAR T fitness. PI3K-AKT-mTOR modulation can restrain terminal differentiation during culture,^99^^,^^105^ whereas PGC-1α engineering directly augments mitochondrial capacity.^135^ TOX/NR4A editing and cytokine-armored CARs act more directly on exhaustion programs or extrinsic TME signals.^40^^,^^41^^,^^42^^,^^43^^,^^113^ SCFAs differ in providing a reversible, non-genetic way to couple substrate metabolism with chromatin regulation during manufacturing. Their most plausible role is therefore as a manufacturing adjunct whose added value should be tested against, and eventually combined with, these other approaches.

### Implications for *in vivo* and *in situ* CAR T generation

CAR T generation is beginning to move out of the manufacturing facility and into the patient. Antigen receptor mRNA delivered by polymer nanocarriers, implantable bioinstructive scaffolds, and CD8-targeted lipid nanoparticles carrying anti-CD19 CAR mRNA have each produced functional CAR T cells without leukapheresis or culture.^143^^,^^144^^,^^145^ First-in-human treatment of relapsed or refractory multiple myeloma with an *in vivo* generated B-cell maturation antigen (BCMA) CAR has also been reported.^146^ This shift raises a direct question for the framework proposed here: does the hypothesis still have meaning when the *ex vivo* expansion culture it is built around disappears?

The framework developed here concerns when the metabolic and chromatin state of a CAR T cell should be set. The period in which a T cell acquires the CAR and undergoes its first rounds of proliferation is also the period in which oxidative capacity, TCF-1-associated accessibility, and vulnerability to TOX-driven exhaustion remain most tractable.^109^^,^^110^^,^^111^^,^^118^^,^^119^ *In vivo* generation relocates this window to lymphoid tissue and the tumor site. The biological premise therefore remains relevant; what changes is the ability to control dose, timing, target population, and washout.

Host SCFA status may matter more when CAR T cells are generated *in vivo*. These platforms introduce the CAR into endogenous T cells whose metabolic and chromatin states have already been shaped by diet, microbiota composition, prior therapy, and antibiotic exposure. No culture step standardizes that starting material, and some current approaches also omit lymphodepleting chemotherapy.^145^^,^^146^ Clinical observations are consistent with this concern: lower pre-infusion serum butyrate was associated with poorer progression-free survival,^101^ while antibiotic-associated loss of microbial metabolic output correlated with poorer CAR T outcomes.^102^ Both measurements were made in the host, making them directly relevant to platforms in which CAR expression and early expansion occur there.

The sequential butyrate-to-pentanoate protocol, by contrast, does not transfer. It depends on four things that only *ex vivo* culture could supply: a defined concentration at the cell, a defined order of exposure, restriction of exposure to the intended population, and a washout before release. Oral or dietary supplementation provides none of these reliably, since colonic production and hepatic first-pass extraction leave peripheral concentrations variable and largely outside operator control. Systemic exposure also reaches cells the protocol was never intended to touch, promoting Foxp3 induction in CD4^+^ T cells, limiting natural killer (NK) effector function, and blunting the antitumor effect of checkpoint blockade in tumor-bearing hosts.^23^^,^^104^^,^^105^^,^^106^ The CD4^+^ compartment is the specific concern, since the CD8^+^ pre-enrichment step available during manufacturing has no *in vivo* equivalent. Receptor-targeted delivery contains part of this problem on the vector side, in that CD8-restricted nanoparticles confine CAR expression to the intended subset,^145^ but it does nothing about systemic SCFA exposure itself. Sequential conditioning as described here should therefore be read as specific to *ex vivo* manufacturing.

*In vivo* generation therefore changes how the framework is applied rather than making SCFA biology irrelevant. Host SCFA status becomes a measurable variable, and its effect on the emerging CAR T population can be tested in immunocompetent models using dietary fiber, defined bacterial consortia, or antibiotic depletion. The timing model predicts that SCFA availability before and during early CAR expression will matter more than sustained exposure after differentiation. Cell-restricted delivery may eventually recover some of the control lost outside culture. SCFA-releasing nano-prodrugs and prebiotic nanoformulations have been developed for other indications,^147^^,^^148^ although co-delivery with a CAR-encoding vector has not yet been tested. For near-term *in vivo* studies, serum and fecal SCFA profiling should therefore be incorporated as biological stratification variables.

## Conclusion and outlook

The evidence reviewed here supports butyrate and pentanoate as candidates for metabolic and epigenetic programming during CAR T manufacturing. SCFAs combine substrate metabolism, class I HDAC inhibition, and receptor signaling in a way that is difficult to reproduce with a single conventional metabolic or epigenetic intervention. Direct CAR T studies now show that butyrate and pentanoate can alter differentiation and antitumor function,^26^^,^^27^^,^^78^^,^^101^ while clinical cohorts link host SCFA status to treatment outcomes.^27^^,^^101^^,^^102^ These findings give the sequential model a credible experimental foundation. Its value will be determined by whether staged exposure preserves progenitor-associated features while adding effector capacity under chronic antigen and metabolic stress.

Translation will require pharmacodynamic and process control. Butyrate and pentanoate each operate within concentration ranges that must be calibrated against expansion yield, viability, phenotype, and potency.^26^^,^^27^^,^^99^^,^^103^ Exposure duration, washout, and residual-metabolite testing should be treated as defined process parameters.^107^^,^^108^ Because CD4+ subsets can respond differently to SCFA-mediated HDAC inhibition, FOXP3+CD25+ frequencies should be prospectively monitored as a candidate safety and product-composition readout.^23^^,^^25^^,^^128^ These measures should be built into good manufacturing practice (GMP)-compatible batch-release criteria, and donor-to-donor and batch-to-batch reproducibility should be established before scale-up. For *in vivo* CAR T generation, the question shifts from transferring an *ex vivo* protocol to measuring host SCFA status and developing controlled local delivery. The current evidence spans selected hematologic and solid-tumor models, but independent testing across donors, CAR designs, and immunocompetent solid-tumor systems remains necessary.

The immediate priority is a matched comparison of each single agent, simultaneous exposure, and both sequence orders under the same manufacturing conditions. This experiment should determine whether early butyrate-associated metabolic and chromatin features persist after pentanoate exposure and whether the sequence improves rechallenge responses beyond the better single agent. Testing both CD28- and 4-1BB-based CARs will establish how baseline costimulatory signaling modifies the response. If staged SCFA exposure provides a distinct benefit, it can then be combined with complementary approaches such as TOX-directed editing, armored CAR designs, or manipulation of ACSS2 and ACLY.^124^ This path preserves the central premise of SCFA conditioning while setting a clear experimental standard for its development.

## Acknowledgments

This research was supported by the 10.13039/501100012166National Key Research and Development Program of China (grant number 2021YFA1100800 to C.-W.D.), the Major Scientific Research Program for Young and Middle-aged Health Professionals of Fujian Province, China (grant number 2023ZQNZD012).

## Author contributions

M.-Y.M. and C.-X.Z. conducted the literature search, prepared the figures, and drafted most of the manuscript; X.-Y.S. contributed to some of the text; C.-W.D. and J.-M.Z. conceived of the general scope and conceptual framework of the review and supervised the writing.

## Declaration of interests

The authors declare no competing interests.
